# Impact of Beamforming on the Path Connectivity in Cognitive Radio Ad Hoc Networks

**DOI:** 10.3390/s17040690

**Published:** 2017-03-27

**Authors:** Le The Dung, Tran Dinh Hieu, Seong-Gon Choi, Byung-Seo Kim, Beongku An

**Affiliations:** 1 Department of Radio and Communication Engineering, Chungbuk National University, Cheongju City, Chungbuk 28644, Korea; dung.t.le@ieee.org; 2 Department of Electronics and Computer Engineering in Graduate School, Hongik University, Sejong City 30016, Korea; trandinhhieu1989@gmail.com; 3 Department of Computer and Information Communication Engineering, Hongik University, Sejong City 30016, Korea; jsnbs@hongik.ac.kr (B.-S.K.); beongku@hongik.ac.kr (B.A.)

**Keywords:** cognitive radio ad hoc networks, directional antenna, uniform linear array antenna, uniform circular array antenna, beamforming scheme, path connectivity

## Abstract

This paper investigates the impact of using directional antennas and beamforming schemes on the connectivity of cognitive radio ad hoc networks (CRAHNs). Specifically, considering that secondary users use two kinds of directional antennas, i.e., uniform linear array (ULA) and uniform circular array (UCA) antennas, and two different beamforming schemes, i.e., randomized beamforming and center-directed to communicate with each other, we study the connectivity of all combination pairs of directional antennas and beamforming schemes and compare their performances to those of omnidirectional antennas. The results obtained in this paper show that, compared with omnidirectional transmission, beamforming transmission only benefits the connectivity when the density of secondary user is moderate. Moreover, the combination of UCA and randomized beamforming scheme gives the highest path connectivity in all evaluating scenarios. Finally, the number of antenna elements and degree of path loss greatly affect path connectivity in CRAHNs.

## 1. Introduction

Recent technological advances have results in the development of wireless ad hoc networks. These networks are composed of devices that are self-organizing and can be deployed without infrastructure support. Due to the proliferation of wireless devices, the industrial, scientific, and medical (ISM) bands are getting congested. Therefore, the efficiency of spectrum usage has become a major concern. To maximize the benefit of usable radio spectrum, spectrum management is needed. However, recent studies highlight that a large percentage of licensed band remains unused because those spectrum bands are allocated through static assignment policies but only used in bounded regions or over a limited period of time. To deal with such bandwidth scarcity and inefficient bandwidth usage, the concept of *cognitive radio* (CR) has been recognized in [1] as an effective method. Specifically, in a CR network, a secondary network is overlaid with a primary network, secondary users (SUs) detect and utilize temporarily unused frequency bands of primary users (PUs) without causing interference to primary users.

Connectivity is a fundamental property of ad hoc networks (AHNs). The connectivity of CRAHNs is different and more challenging compared with conventional AHNs because secondary users have to opportunistically utilize the licensed spectrum band of primary users. There have been extensive studies on the connectivity of conventional AHNs [2,3,4,5,6]. Particularly, for nodes with omnidirectional antennas and disk transmission range $r_{0}$, the authors in [2] present a framework for calculation of stochastic connectivity of wireless multi-hop AHNs. Then, the impact of critical transmission range, i.e., the minimum common value of the nodes’ transmitting range that produces a connected communication graph, on connectivity of AHNs in the presence of node mobility, is studied in [3]. For a more accurate modeling of the wireless channel, the connectivity of AHNs under the effects of log-normal shadowing, Rayleigh fading, and Nakagami-*m* fading is analyzed in [4,5,6], respectively. It is shown that fading environment helps to increase the connectivity of AHNs. Topological connectivity of AHNs, where wireless nodes deploy directional antennas, is examined in [7,8,9,10,11]. The authors conclude that the application of the beamforming antenna leads to significant improvement in the multi-hop connectivity of AHNs.

Recently, researchers have been attracted to investigating the connectivity of CRAHNs. In [12], the authors introduce a metric called *algebraic connectivity*. Firstly, a connection graph of CRAHNs is built. Then, the second smallest eigenvalue of the Laplacian of this graph is used to evaluate the influence degree of PUs on the communication probability among SUs. Local connectivity, i.e., node degree and isolation probability of SU with Gaussian noise and path loss model are investigated in [13] by employing stochastic geometry and probability theory. Using percolation theory, the authors in [14] give the lower and upper limits of the percolation area within which the secondary network percolates while the outage probability of PU is kept below a tolerable threshold. The analysis of connectivity of CRAHNs in non-shadowing and log-normal shadow fading environment is presented in [15,16], respectively. In these works, it is assumed that all PUs and SUs are equipped with ominidirectional antennas. Although the effect of beamforming on the connectivity of AHNs was studied in the literature [7,8,9,10,11] and the applications of using directional antennas in AHNs and CRAHNs are presented in [17,18,19], to the best of our knowledge, no works on evaluating the connectivity of CRAHNs with beamforming have been carried out.

**Figure 1:**
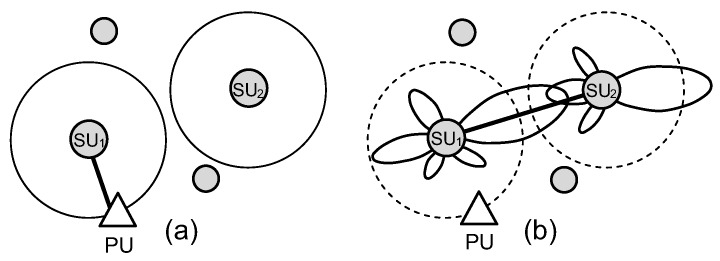
Difference in the network connectivity of CRAHNs with (**a**) omidirectional antenna and (**b**) directional antenna.

Figure 1 shows how the directional antenna affects the network connectivity and make comparisons with the scenario in which omnidirectional antenna is used in the cognitive environment. Specifically, the difference in the possibility of a wireless link establishment between two SUs when they are equipped with omnidirectional antennas and directional antennas are illustrated in Figure 1a,b, respectively. As we can see in Figure 1a, $SU_{1}$ is not allowed to transmit packets to $SU_{2}$ because $SU_{1}$ interferes with PU. Moreover, due to using omnidirectional antenna in both SUs, the transmission and reception antenna gains in the $SU_{1}$–$SU_{2}$ direction are not high enough to create a wireless connection between these two SUs. However, in Figure 1b, where both $SU_{1}$ and $SU_{2}$ use directional antennas, $SU_{1}$ can operate normally thanks to the antenna gain of $SU_{1}$ in the $SU_{1}$–PU direction is very small. In addition, $SU_{1}$ can communicate with $SU_{2}$ because the main beam of $SU_{1}$ antenna stares at $SU_{2}$, making the signal attenuation of wireless link $SU_{1}$–$SU_{2}$ lower than a threshold.

This observation motivates us to evaluate whether beamforming always benefits the connectivity of CRAHNs like the case of AHNs. If not, then when, how, and why the usage of directional antennas and beamforming schemes increases or decreases the connectivity of CRAHNs by comparing it to the CRAHNs with ominidirectional antennas.

The main contributions of this paper are:

1. We examine the combined influence of different antenna types and beamforming schemes on the path connectivity of CRAHNs. Particularly, we consider how SUs equipped with two popular directional antennas, i.e., uniform linear array (UCA) and uniform circular array (UCA) antennas, communicate with each other by using two simple and efficient beamforming schemes, i.e., randomized beamforming and center directed beamforming. Especially, we show that, in contrast to AHNs, using beamforming in CRAHNs does not always improve network connectivity. To be more specific, in all evaluating scenarios, only the UCA antenna gives higher path connectivity than omnidirectional antennas.
2. We show that the influence of beamforming on path connectivity greatly depends on the degree of channel path loss. Specifically, when path loss exponent α = 3, path connectivity remains stable, but the maximum values are lower than that when α = 2.
3. We find that the number of antenna elements of directional antennas significantly affects path connectivity. For each type of directional antenna, the number of antenna elements, at which the highest path connectivity is obtained, is different.

The results in this paper provide insights into how beamforming changes the connectivity characteristics of CRAHNs under path loss and Rayleigh fading and helps network designers to select the appropriate directional antenna and beamforming scheme in order to maximize network performance of CRAHNs.

The rest of this paper is organized as follows. Section 2 introduces the system model including antenna model, network model, and wireless link used in this paper. Section 3 presents the characteristics of two different beamforming schemes and our motivations behind the investigation of the influence of beamforming on the communication probability among SUs in CRAHNs. The results and discussions are presented in Section 4. Finally, the paper is concluded in Section 5.

## 2. System Model

### 2.1. Antenna Model

In this paper, we consider two directional antennas, i.e. Uniform Linear Array (ULA) and Uniform Circular Array (UCA) antennas. The ULA comprises of antenna elements separated a distance of Δ along the line, whereas in UCA, the antenna elements are arranged on a circle with radius of *r*.

Since each single element is ominidirectional antenna, $E_{s}\left(\theta ,\phi \right)=E_{0}$, where $E_{s}\left(.\right)$ refers to the far-zone electric field of each antenna element, θ is the polar angle and ϕ is the azimuth angle in polar coordinates, respectively.

For an array antenna, its far-zone electric field is calculated by multiplying the electric field of single element by the array factor of that array, that is,

(1) $$E\left(array\right)=\left[E_{S}\left(\theta ,\phi \right)\right]\times \left[AF\left(\theta ,\phi \right)\right].$$

Then, the gain of an array antenna can be expressed as a function of its array factor, namely

(2) $$G\left(\theta ,\phi \right)=\frac{{\left|AF(\theta ,\phi )\right|}^{2}}{\frac{1}{4\pi }\int_{0}^{2\pi }\int_{0}^{\pi }{\left|AF(\theta ,\phi )\right|}^{2}\sin\theta d\theta d\phi },$$

where AF(θ,ϕ) is the array factor of array antenna.

The array factor of ULA antenna is given by [20,21]

(3) $$AF\left(\theta ,\phi \right)=\sum_{m=1}^{M}e^{j(m-1)\psi },$$

where *M* represents the number of antenna elements, ψ=kΔcosϕ+β, k=2π/λ is the wave number, and Δ and β are the distance and phase excitation difference between antenna elements.

Since the physical center of the ULA antenna is often chosen as the reference point when calculating its array factor; thus, Equation (3) becomes

(4) $$AF\left(\theta ,\phi \right)=\frac{\sin(\frac{M}{2}\psi )}{\sin(\frac{1}{2}\psi )}.$$

By plugging Equation (4) into Equation (2), we can obtain the antenna gain of ULA for different values of antenna elements *M* and azimuthal angle ϕ.

The array factor of UCA is expressed as [20,21]

(5) $$AF\left(\theta ,\phi \right)=\sum_{m=1}^{M}I_{m}e^{j[kr\;\sin\theta \cos\left(\phi -\phi_{m}\right)+\alpha_{m}],}$$

where *r* is the radius of the circle formed by the antenna elements, $\phi_{m}=2\pi m/M$, $I_{m}$, and $\alpha_{m}$ refers to the angular position, amplitude excitation, and phase excitation of *m*-th element, respectively.

To have the peak of main beam stare at ($\theta_{0},\phi_{0}$) direction, the phase excitation of the *m*-th element is selected such that

(6) $$\alpha_{m}=-kr\sin\theta_{0}\cos\left(\phi_{0}-\phi_{m}\right).$$

Consequently, the array factor of UCA antenna can be rewritten as

(7) $$AF\left(\theta ,\phi \right)=\sum_{m=1}^{M}I_{0}e^{kr[j\;\sin\theta \cos\left(\phi -\phi_{m}\right)-\sin\theta_{0}\cos\left(\phi_{0}-\phi_{m}\right)]}.$$

Similarly, by substituting Equation (7) into Equation (2), the gain of the UCA antenna can be calculated for different values of antenna elements *M* and azimuthal angle ϕ.

In this paper, since the connectivity of two dimensional CRAHNs is studied, we only consider the azimuthal plane by setting $\theta =\theta_{0}=\pi /2$.

**Figure 2:**
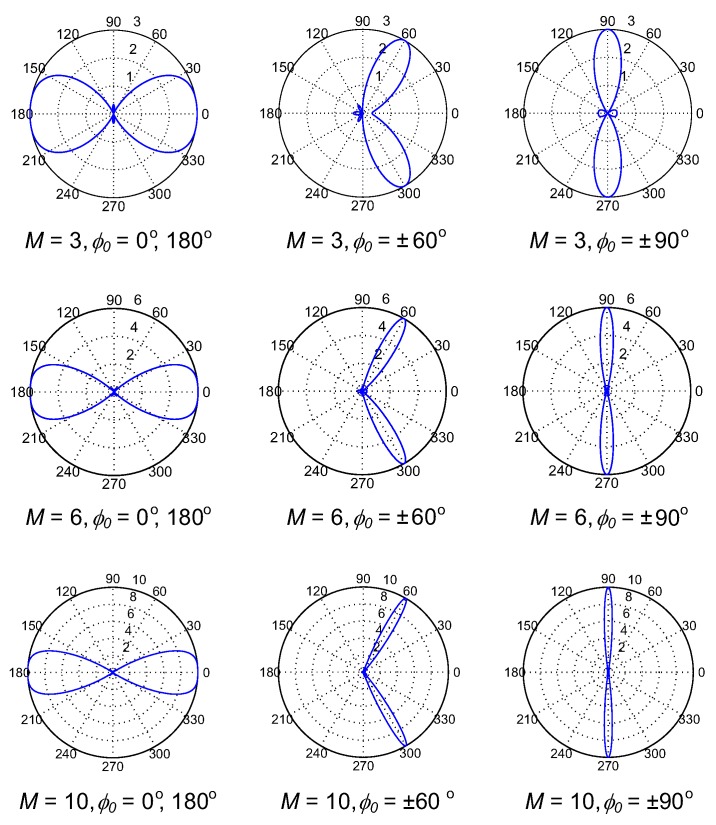
Gain patterns of ULA as the numbers of antenna elements and main beam directions are varied.

**Figure 3:**
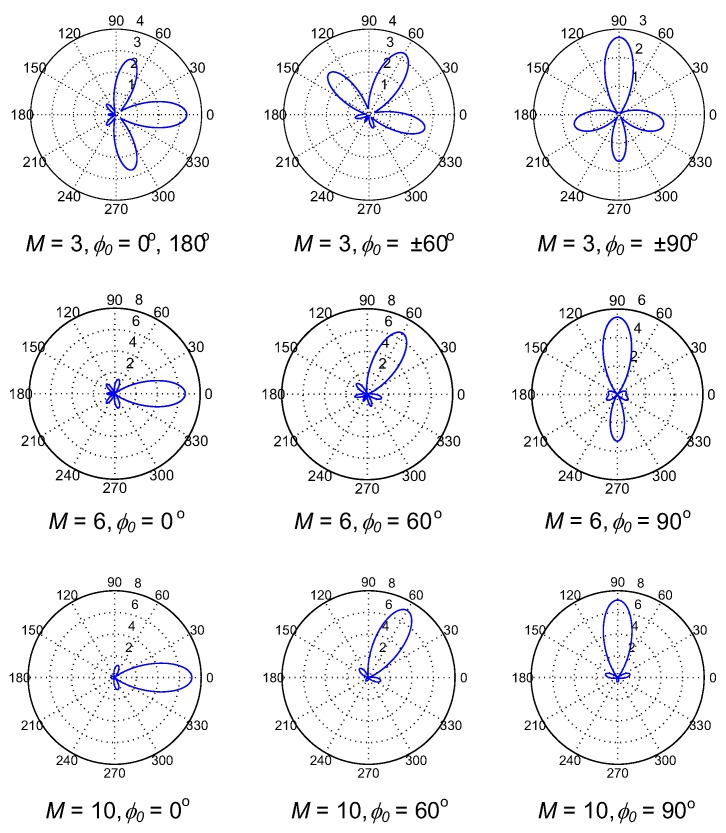
Gain patterns of UCA as the numbers of antenna elements and main beam directions are varied.

The gain patterns of ULA and UCA antennas are plotted in Figure 2 and Figure 3, respectively, as the numbers of antenna elements and main beam directions are varied. As we can see in Figure 2, the gain pattern of ULA has two main beams and the peak values of main beams are independent of their directions, i.e., they are always equal to the number of antenna elements *M*. Moreover, the beam width reduces as the number of antennas gets higher. In contrast, the gain pattern of the UCA has only a single main beam, whose width is almost independent of its direction and the peak is not equal to *M*. These features will result in the differences in connection probability among SUs in CRAHNs when these two directional antennas are employed. We will discuss these differences in detail in Section 4.

### 2.2. Network Model

**Figure 4:**
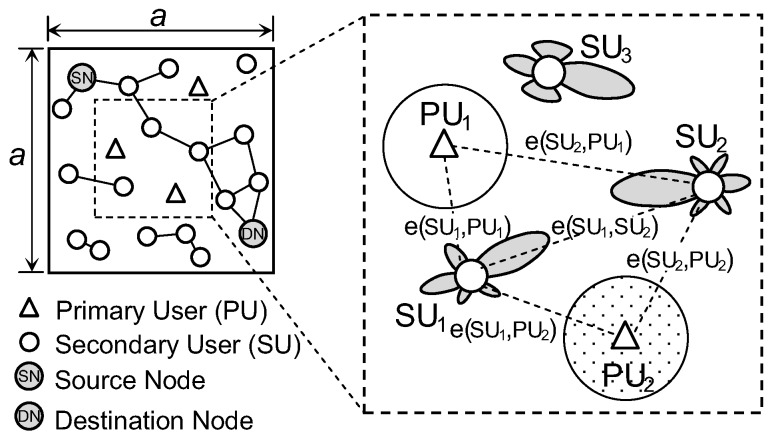
The network model of CRAHNs where SUs employ directional antennas and PUs use omnidirectional antennas.

In this paper, we assume that PUs use omnidirectional antennas while SUs are equipped with one of two kinds of directional antennas, i.e., uniform linear array (ULA) and uniform circular array antennas as in Figure 4. These directional antennas were widely used in evaluating the connectivity of conventional ad hoc networks with beamforming [7,8]. All PUs and SUs share one licensed spectrum band and are randomly located in the network area of a×a according to the Poisson point process. The operation of PUs on a licensed spectrum band is associated with ON and OFF states where the number of times, *x*, that PU occupies a licensed spectrum in a time unit is determined by Poisson distribution with active rate $\lambda_{P}$ i.e.,

(8) $$P_{X}\left(x\right)=\frac{\lambda_{P}^{x}}{x!}e^{-\lambda_{P}}.$$

The communications between a specific SU and other SUs occur when all of its neighboring PUs are in the OFF state, i.e., the overlay transmission mode [15,16].

### 2.3. Wireless Link Model

The wireless link between two nodes are described as follows. One node transmits signal with power $P_{t}$ that is received by the other node with power $P_{r}$. We assume that large scale path loss and small scale Rayleigh fading contribute to the fluctuation in signal power. Specifically, path loss decreases the signal power by a path loss exponent α of the environment (e.g., α≈2 represents free space, and α≈2.7 to 5 represents urban area). Whereas, Rayleigh fading channel affects the probability density function of received signal amplitude, which is expressed as [22]

(9) $$f_{U}\left(u\right)=\frac{2u}{\Omega }\exp\left(-\frac{u^{2}}{\Omega }\right),$$

where *u* is Rayleigh distributed random variable and $E(u^{2})=\Omega$ is the average value of received signal power.

Hence, the received power $P_{r}$ under the combined impacts of large scale path loss, small scale Rayleigh fading, and antenna gains is given by [22,23]

(10) $$P_{r}=u^{2}\frac{1}{d^{\alpha }}G_{t}G_{r}P_{t}.$$

It should be noticed that when nodes are equipped with directional antennas, the antenna gains of transmitter $G_{t}$ and receiver $G_{r}$ are random variables instead of $G_{t}$ = $G_{r}$ = 1 when omnidirectional antennas are used.

From Equation (10), the signal power attenuation is given by

(11) $$\gamma \left(d\right)=\frac{P_{t}}{P_{r}}=\frac{d^{\alpha }}{u^{2}G_{t}G_{r}},$$

and is expressed in the unit of dB as

(12) $$\gamma {\left(d\right)}_{dB}=10\log_{10}\left(\frac{P_{t}}{P_{r}}\right)=\alpha 10\log_{10}\left(d\right)-10\log_{10}\left(u^{2}G_{t}G_{r}\right).$$

Thus, when the signal power attenuation between two nodes separated at a distance *d* is less than a specific threshold $\gamma_{th}$, i.e., $\gamma \left(d\right)\leq \gamma_{th}$, they are connected via a wireless link.

## 3. The Impact of Beamforming on the Connectivity of Cognitive Radio Ad Hoc Networks

We study the influence of directional antenna and beamforming schemes on the path connectivity between two random SUs in CRAHNs. Two aforementioned array antennas are used in two beamforming schemes to evaluate in what conditions beamforming schemes benefit the connectivity of CRAHNs.

**Figure 5:**
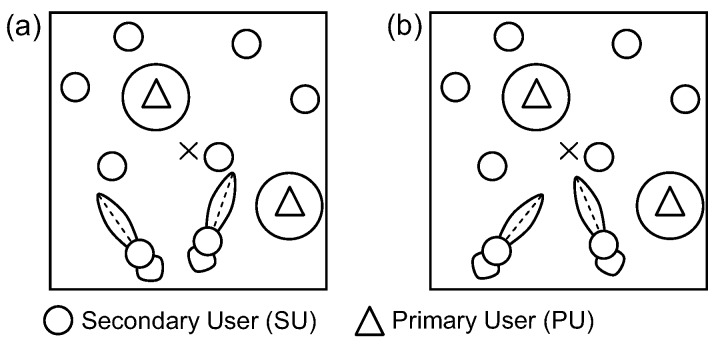
Illustration of two beamforming schemes used to evaluate the connectivity of CRAHNs: (**a**) randomized beamforming; (**b**) center-directed beamforming.

Figure 5 illustrates the two beamforming schemes, i.e., randomized beamforming and center-directed beamforming, used to examine the communication possibilities among SUs in CRAHNs. The features of these schemes are briefly described as follows.

- **Randomized beamforming**: This beamforming scheme is considered as the simplest one, i.e., each node chooses the direction of its main beam from [0,2π] based on uniform random distribution, and completely independent of other nodes.
- **Center-directed beamforming**: According to this beamforming scheme, it is required that all nodes know the center of network area. Then, they point their main beams toward the network center.

Because of no additional signaling for the information of neighboring location and beam direction, these beamforming schemes are efficient in CRAHNs where the routing and spectrum sensing process already employ a lot of control messages. Investigating the effects of the randomized beamforming scheme and the center-directed beamforming scheme on the connectivity of ad hoc networks is presented in [7,8], respectively.

We are interested in the questions: does a directional antenna always benefit the connectivity of CRAHNs? What kinds of antennas and beamforming schemes benefit the connectivity of CRAHNs? Moreover, since the number of antenna elements remarkably changes the antenna gain pattern, we also investigate the optimal number of antenna elements that provides the highest connectivity.

## 4. Experimental Results and Discussions

In this section, we conduct Monte Carlo simulation by running MATLAB (R2016a, MathWorks Inc., Natick, MA, USA) codes on a computer to investigate the effect of beamforming on the path connectivity in CRAHNs. Similar to previous works [12,13,14,15,16], we model CRAHNs as random graphs. Initially, a square area with size a×a is created. Next, $N_{S}$ secondary users and $N_{P}$ primary users are randomly placed in this square area by using the Poisson point process. The active state of each PU is determined by Poisson distribution with active rate $\lambda_{P}$. The communication links among nodes are affected by large scale path loss, small scale Rayleigh fading, and antenna gains. For each network topology, new random locations of PUs and SUs and random active states of PUs are used. The simulation results are obtained by averaging the connectivity outcomes of 10,000 network topology trials. Simulation time varies from 0.9 h to 8.7 h, depending on the settings of node density and antenna type used in each evaluating scenario. To measure the level of connectivity, we consider the path connectivity $P_{path}$. It is defined as the probability that two randomly selected SUs in CRAHNs are connected via a multi-hop path or direct link and is calculated as the statistical average percentage of connected path as [24]

(13) $$P_{path}=\frac{\#\;\mathrm{connected}\;\mathrm{paths}}{\#\;\mathrm{network}\;\mathrm{topology}\;\mathrm{trials}}.$$

We will compare the connectivity performance $P_{path}$ of four combinations of directional antennas and beamforming schemes (i.e., ULA-random, ULA-center, UCA-random, and UCA-center) under the effect of node density, the configuration of directional antenna, the average active rate of primary user, and path loss level. Path connectivity of CRAHNs with omnidirectional antenna (corresponds to the number of antenna elements M=1) is also investigated. We aim to intensively study how the path connectivity is affected under various combinations of these network parameters. Similar evaluating scenarios can be found in [8] for AHNs and in [16] for CRAHNs.

### 4.1. Effect of SU Density

**Figure 6:**
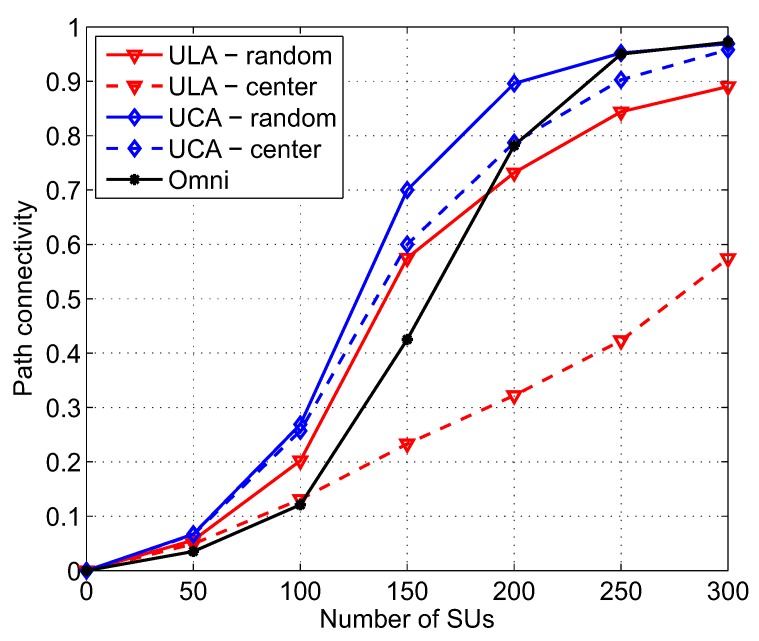
Path connectivity corresponding to different kinds of antennas and beamforming schemes as a function of the number of SUs; *a* = 500 m, *M* = 6, $N_{P}$ = 3, $\lambda_{P}$ = 0.1, α = 3, $\gamma_{th}$ = 50 dB.

Figure 6 shows path connectivity versus SU density with different combinations of antenna types and beamforming schemes compared with omnidirectional antennas. We can see that, for a low number of SUs, i.e., $N_{S}$ ≤ 50, the path connectivity is low, and there is no significant difference between the two directional antenna types and omnidirectional antennas because the node density is not high enough to ensure that an SU can always find at least one neighboring SU to communicate with, regardless of antenna types and beamforming schemes. As the number of SUs gets higher, the levels of path connectivity of all five scenarios significantly increase. UCA-random mostly outperforms omnidirectional antennas. However, ULA-center, ULA-random, and UCA-center only give higher path connectivity when $N_{S}$ ≤ 100, $N_{S}$ ≤ 170, and $N_{S}$ ≤ 200, respectively. The reasons can be explained as follows. Compared with random beamforming scheme, the center beamforming scheme may not be good for networks where nodes are uniformly distributed because the main beams of all nodes stare at the network center, resulting in less connections among many nodes which are far from the network center. Regarding to directional antennas, UCA is more suitable for cognitive environment because its gain pattern consists of only one main beam whose peak gain is high and beamwidth is stable while side beams are very small. Thus, SU with UCA can avoid interfering with PUs more efficiently. In contrast, the gain pattern of ULA has two main beams where the angle between them is two times that of the main beam angle. To prevent interference with PUs, there are more possibilities that SUs with ULAs are not allowed to communicate with each other. When $N_{S}$ > 170, path connectivity of an omnidirectional antenna is almost equal to that of UCA-random because the number of SUs is high enough so that any SU has many neighboring SUs. Even if some of its neighbors are not allowed to communicate because of active PUs, there are still a lot of other available SUs that enable the establishment of communication links. Thus, the benefit of UCA-random on path connectivity is insignificant in a dense network. Since ULA-center is constituted by two unfavorable components for a cognitive environment as mentioned above, i.e., ULA antenna and center-directed beamforming scheme, it gives much lower path connectivity compared with the others.

### 4.2. Effect of PU Density

**Figure 7:**
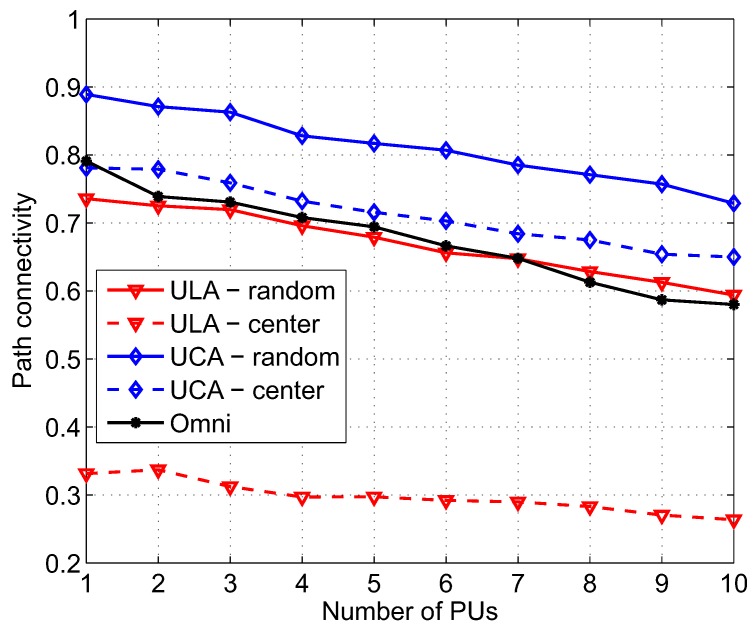
Path connectivity corresponding to different kinds of antennas and beamforming schemes as a function of the number of PUs; *a* = 500 m, *M* = 6, $N_{S}$ = 200, $\lambda_{P}$ = 0.5, α = 3, $\gamma_{th}$ = 50 dB.

Figure 7 shows path connectivity versus node density of PU when different combinations of antenna types and beamforming schemes are used. Network size, *a*, and number of SUs, $N_{S}$, are fixed at 500 m and 200, respectively, while the number of PUs, $N_{P}$, in the network increases from 1 to 10. When there are more PUs in the network area, from the viewpoint of spatial spectrum occupation, the available network area for routing among SUs is reduced. Consequently, the degrees of path connectivity of all five scenarios are reduced. However, the UCA-random still outperforms the others, i.e., 0.73 compared to 0.65, 0.59, and 0.26 of UCA-center, ULA-random, and ULA-center, respectively, when $N_{P}$ = 10.

### 4.3. Effect of the Average Active Rate of PU

**Figure 8:**
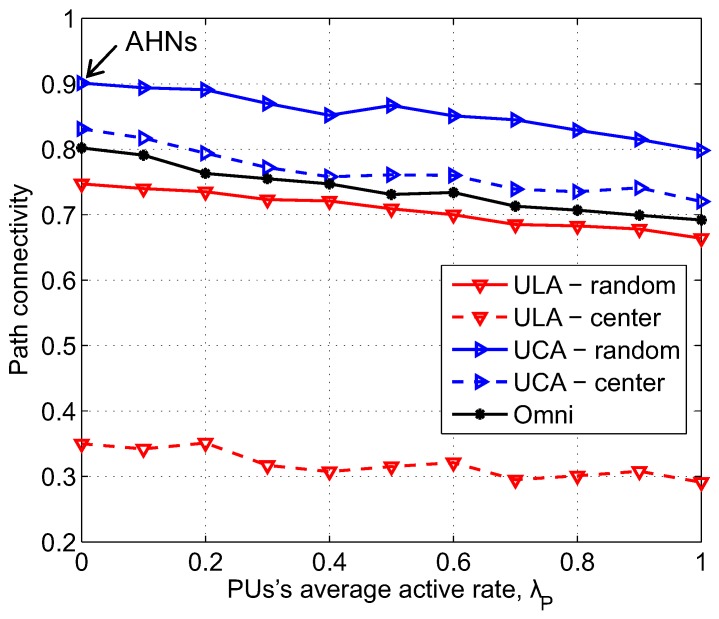
Path connectivity corresponding to different kinds of antennas and beamforming schemes as a function of the average active rate of PU; *a* = 500 m, *M* = 6, $N_{S}$ = 200, $N_{P}$ = 3, α = 3, $\gamma_{th}$ = 50 dB.

The effect of the average active rate of PU, $\lambda_{P}$, on path connectivity is depicted in Figure 8. The average active of PU range varies from 0 to 1 to reflect low and high occupation of licensed channel. We should mention that when $\lambda_{P}$ = 0, i.e., there is no influence of the primary network on the secondary network, CRAHNs can be considered as standalone AHNs. As shown in Figure 8, similar to the impact of PU density, increase in the average active rate of PU also reduces the available network area for routing among SUs, and, thus, results in the decrease in successful path establishment probability. Particularly, in this evaluating scenario, when $\lambda_{P}$ rises from 0 to 1, path connectivity corresponding to UCA-random, UCA-center, ULA-random, ULA-center, and Omni reduces from 0.91, 0.83, 0.75, 0.35, and 0.81 to 0.79, 0.72, 0.66, 0.29, and 0.68, respectively. From these obtained results, it can be seen that ULA antenna gives lower path connectivity than omnidirectional antenna in the whole range of $\lambda_{P}$. Especially, in the case of ULA-center, path connectivity is remarkably low. The UCA outperforms other antennas. Again, the combination of UCA antenna and randomized beamforming scheme provides the highest path connectivity. In addition, the gap in path connectivity correlating with UCA-random is significant compared with other combinations of directional antenna and beamforming scheme.

### 4.4. Effect of the Configuration of Directional Antenna

**Figure 9:**
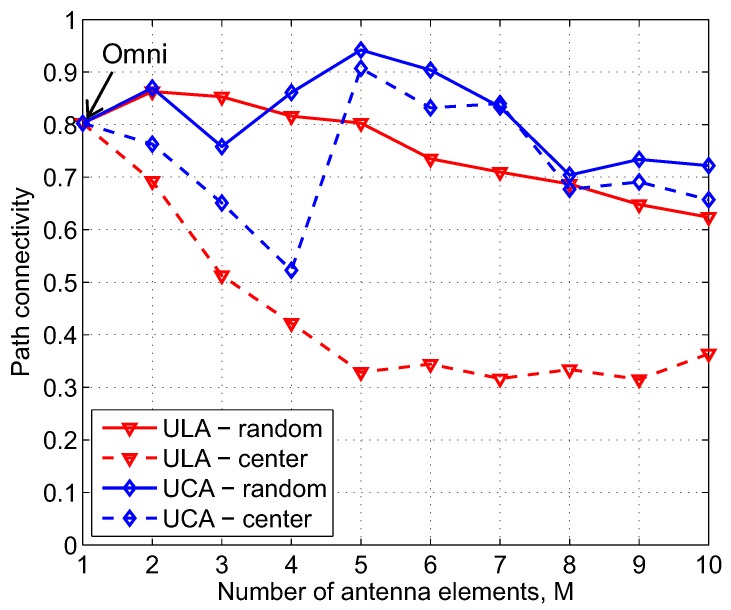
Path connectivity corresponding to different kinds of antennas and beamforming schemes as a function of the number of antenna elements; *a* = 500 m, $N_{S}$ = 200, $N_{P}$ = 3, $\lambda_{P}$ = 0.1, α = 3, $\gamma_{th}$ = 50 dB.

As observed in Figure 2 and Figure 3, the number of antenna elements, *M*, of directional antennas greatly affects their gain patterns, which definitely influences the overall network connectivity. Figure 9 shows the relation of path connectivity when *M* increases from 1 to 10. It should be noted that *M* = 1 corresponds to the omnidirectional antenna. As can be seen in Figure 9, path connectivity is greatly varied as the number of antenna elements changes. When *M* ≤ 3, the levels of path connectivity of UCA-random and UCA-center are lower than ULA-random because, when *M* is small, the gain pattern of UCA has more high-gain side beams compared with ULA. ULA-center has the lowest path connectivity because, with two symmetric main beams of ULA, using the centralized beamforming scheme may remarkably create a high possibility of interfering with PUs. As *M* increases, the gain pattern of UCA now consists of only one high-gain main beam and considerably small-gain side beams, which is efficient in cognitive networks. Generally, UCA-random yields better path connectivity than the others, and reaches the highest value of 0.94 when *M* = 5.

### 4.5. Effect of Path Loss

**Figure 10:**
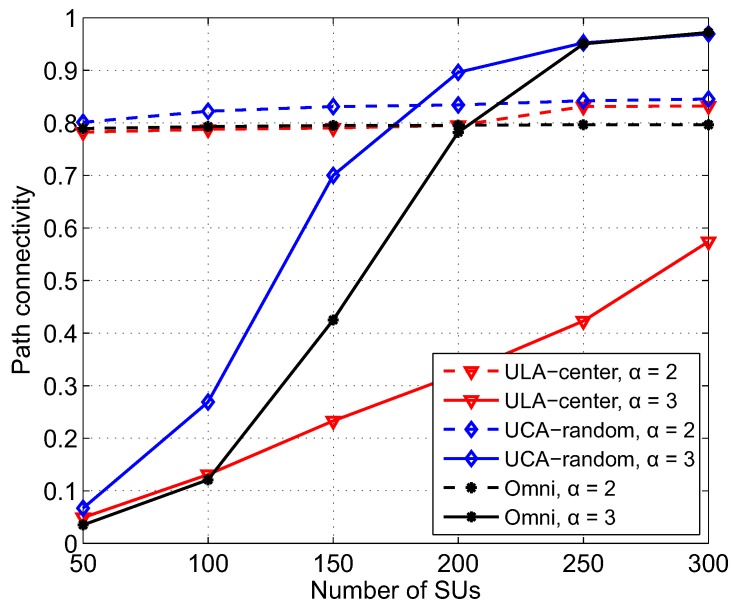
Path connectivity corresponding to different kinds of antennas and beamforming schemes as a function of the number of SUs with path loss exponent α = 2 and 3, *a* = 500, *M* = 6, $N_{S}$ = 200, $N_{P}$ = 3, $\lambda_{P}$ = 0.1, $\gamma_{th}$ = 50 dB.

We now evaluate the combined effect of beamforming and the degree of path loss on path connectivity in CRAHNs. From Figure 10, we can see that path loss exponent α remarkably impacts the connectivity. When α = 3, the differences in path connectivities of ULA-center, UCA-random, and omnidirectional antenna are noticeable. The reasons why when α = 3, UCA-random provides the highest path connectivity while ULA-center gives the lowest are explained in the scenario evaluating the effect of SU density. In contrast, when α = 2, the differences in path connectivity of ULA-center, UCA-random, and omnidirectional antenna are insignificant. It should be noticed that the path connectivity when α = 2 is approximately 0.79 even at a low number of SUs, i.e., $N_{S}$ = 50, compared with that of 0.05 when α = 3. Since lower path loss exponent results in higher received power, the wireless connectivity among SUs is considerably increased. However, at the same time, the interference from SUs to PUs also increased. This will prohibit SUs from communicating with other SUs. Due to these reciprocal effects, path connectivity when α = 2 in all beamforming scenarios remains stable but lower than that when α = 3 even when more SUs are put in the networks. Regardless of path loss exponent α, UCA-random still gives higher path connectivity compared with ULA-center and omnidirectional antenna.

## 5. Conclusions

We have studied the effects of different combinations of directional antennas and beamforming schemes on the path connectivity between two arbitrary SUs in CRAHNs. Particularly, two directional antennas, i.e., ULA and UCA, and two beamforming schemes, i.e., randomized beamforming and center-directed beamforming, are employed. The results on path connectivity of beamforming scenarios are compared with those of omnidirectional antenna to evaluate in what conditions beamforming increases or decreases path connectivity between two arbitrary SUs in CRAHNs. We discover some important and interesting features which can be summarized as follows. First, using beamforming transmission for SUs only shows noticeable benefits in terms of path connectivity of a secondary network when SU density is moderate. Second, the combination of UCA antenna and randomized beamforming provides the highest connectivity compared with others. Third, the number of antenna elements and degree of path loss greatly influence the path connectivity. The results in this paper give insights on how beamforming affects path connectivity in CRAHNs. An important feature is that using any kind of directional antennas does not always provide higher path connectivity compared with ominidirectional antennas. Thus, this paper can help network designers select proper directional antenna configuration and beamforming schemes to achieve the highest path connectivity in CRAHNs. Carrying out our real experiments to measure the path connectivity and comparing the measured results with the simulation results obtained in this paper are considered part of our future work.
